# How to Treat One in a Faint

**Published:** 1891-04

**Authors:** 


### HOW TO TREAT ONE IN A FAINT.

This is something every person should know. First of all loosen every tight thing from around the neck or abdomen ; that is, unfasten the collar from round the neck, and if the patient is a lady cut her stay laces if she wears stays. Allow the person all the fresh air possible, do not crowd around, and if in a crowded place carry the patient out, or to the open window. A fainting person should always be laid flat down on the back, and it greatly aids recovery if the head can be put lower than the body, so that blood goes readily to the brain. The main cause of fainting is that the brain is deprived of blood, and if the head is laid low the brain can get its share again, and so resume its workings. Cold water sprinkled over the face, smelling salts, or burning feathers held to the nose, and fanning the face, all help to restore consciousness. In an ordinary case the person may be allowed to sit up when conscious, and after a little rest resume her way. The custom of giving brandy or other spirits to a person who has fainted is, says Dr. Allinson, a mischievous one ; allow the person to come to, then let her slowly drink a cupful of cold water, and no harm is done. But if brandy is given, the person may pass from one fit to another, or become ill from the drink given. Medicines of any kind are not needed after fainting, only care must be taken to take things quietly for the next few hours. Persons subject to these attacks must keep out of close, hot, and unventilated places, either of devotion or of amusement ; they should not take Turkish baths, nor even hot baths ; in place of the latter they may have a sponge all over with hot water. Tea and coffee must not be drunk by those subject to fainting attacks ; if ladies, they must not wear corsets. Men must not use tobacco in any form, nor drink intoxicants, if subject to these attacks. Heavy and indigestible foods, like pork, veal, ham, etc., must be avoided ; as must heavy work.—*Weekly Echo.*

